# Phosphorylation of CAD1, PLDdelta, NDT1, RPM1 Proteins Induce Resistance in Tomatoes Infected by *Ralstonia solanacearum*

**DOI:** 10.3390/plants11060726

**Published:** 2022-03-09

**Authors:** Prachumporn Nounurai, Anis Afifah, Suthathip Kittisenachai, Sittiruk Roytrakul

**Affiliations:** 1 Innovative Plant Biotechnology and Precision Agriculture Research Group, National Center for Genetic Engineering and Biotechnology, National Science and Technology Development Agency, Pathum Thani 12120, Thailand; 2 Molecular and Applied Microbiology Laboratory, Diponegoro University, Jawa Tengah 50275, Indonesia; anisaf06@gmail.com; 3 Functional Ingredients and Food Innovation Research Group, National Center for Genetic Engineering and Biotechnology, National Science and Technology Development Agency, Pathum Thani 12120, Thailand; suthathip@biotec.or.th

**Keywords:** *Ralstonia solanacearum*, tomato, *Solanum lycopersicon*, phosphorylation, bacterial wilt, defense response, phosphoproteomics

## Abstract

*Ralstonia solanacaerum* is one of the most devastating bacteria causing bacterial wilt disease in more than 200 species of plants, especially those belonging to the family *Solanaceae*. To cope with this pathogen, plants have evolved different resistance mechanisms depending on signal transduction after perception. Phosphorylation is the central regulatory component of the signal transduction pathway. We investigated a comparative phosphoproteomics analysis of the stems of resistant and susceptible tomatoes at 15 min and 30 min after inoculation with *Ralstonia solanacearum* to determine the phosphorylated proteins involved in induced resistance. Phosphoprotein profiling analyses led to the identification of 969 phosphoproteins classified into 10 functional categories. Among these, six phosphoproteins were uniquely identified in resistant plants including cinnamyl alcohol dehydrogenase 1 (CAD1), mitogen-activated protein kinase kinase kinase 18 (MAPKKK18), phospholipase D delta (PLDDELTA), nicotinamide adenine dinucleotide transporter 1 (NDT1), B3 domain-containing transcription factor VRN1, and disease resistance protein RPM1 (RPM1). These proteins are typically involved in defense mechanisms across different plant species. qRT-PCR analyses were performed to evaluate the level of expression of these genes in resistant and susceptible tomatoes. This study provides useful data, leading to an understanding of the early defense mechanisms of tomatoes against *R. solanacearum*.

## 1. Introduction

Plants have developed different sophisticated defense mechanisms to counteract numerous potential pathogens. These defense mechanisms range from basal resistance to inducible resistance called innate immunity. The basal resistance includes constitutively formed physical barriers such as cuticle, wax, and chemical compounds. The innate immunities are divided into two main categories, PAMP-triggered immunity (PTI) and Effector-triggered immunity (ETI) [1,2,3]. PTI is induced by the recognition of pathogen-associated molecular patterns (PAMPs) through pattern recognition receptors (PRR). PAMPs are microbial structural molecules such as flagellins, lipopolysaccharide, Harpin protein, and glycan [4]. The signalings in PTI mediate different pathways including mitogen-activated kinases (MAPKs), Ca^2+^ and H^+^ influx, and accumulation of reactive oxygen species (ROS). Some pathogens inhibit PTI by secreting the effectors into the plant cytoplasm through type three secretion systems (TTSS). To cope with these, plants induce ETI through the recognition of effectors by the intracellular receptor protein NB-LRR. ETI is more race specific and is associated with a hypersensitive response (HR). Both PTI and ETI mediate the production of reactive oxygen species (ROS), reactive nitrogen species such as nitric oxide (NO), the activation of the expression of pathogenesis-related (PR) proteins, and the induction of defense signaling hormones [5,6,7]. The level of the ETI-induced response is thought to be significantly higher than PTI and associated with a hypersensitive response, (HR) [5,8] although some PAMPs such as Hrzp Harpin also induce HR [9] and some ETI responses are very weak. Therefore, the defense responses are not determined by their intensity but by the microbial molecules and their associated receptors. Reaction promptness depends on signaling cascades. Phosphorylation, one of the most studied post-translational modifications (PTMs), is essential for signal transduction during PTI and ETI. It is a reversible process that is catalyzed by kinase and phosphatase. Protein kinases add phosphate groups to proteins leading to a conformational change in the structure of the proteins followed by changes in localization, interaction, and activation. Via an investigation of the putative substrates of nine protein kinases that function in plant biotic and abiotic stress response, 5075 putative target sites were identified using a kinase-assay-linked phosphoproteomics (KALIP) approach [10]. Phosphorylation activates downstream proteins and regulates many cellular processes including cell metabolism, cell growth, and the cell cycle [11,12,13,14]. Several studies have shown that phosphorylation participates in the signaling cascade during plant defense response and enables the plant to react against the pathogen in time [15,16,17]. In *Arabidopsis* cells treated with elicitors, flg22, and xylanase, quantitative phosphoproteomics was investigated and differentially phosphorylated sites were identified in 76 membrane-associated proteins using a mass spectrometry-based method [18]. In rice, a large scale of phosphoproteins and phosphosites was identified to reveal the signal transduction during *Xanthomonas oryzae* pv. *oryzae* (Xoo) infection [19]. Phosphoproteomic analysis was performed to study the effects of salicylic acid (SA) on the rice defense responses against blast disease caused by *Magnaporthe oryzae * [20]. Proteomic and phosphoproteomic analysis of plasma membrane and cytosolic proteins of the rice leaves was employed to reveal the function of MSp1, a *Magnaporthe oryzae* secreted protein to the defense signaling [21]. In maize, the levels of 244 phosphoproteins were significantly changed after SA treatment [22].

Bacterial wilt caused by *Ralstonia solanacearum* is economically one of the most important bacterial phytopathogens. It is a Gram-negative, aerobic rod bacterium classified in the β-subdivision of Proteobacterium and has a worldwide distribution with a large expanding host range of more than 200 plant species such as tomato, potato, tobacco, banana, peanut, and papaya [23,24]. The bacteria invade the plant through a wound or natural opening resulting from the emergence of the lateral root. They spread through the root cortex and the xylem vessels where they can move to the upper parts of the plant via the plant’s transpirational flow [25]. In the xylem, they can reach up to 10^10^ cells per cm of stem resulting in the blockage of the vessels, followed by wilting of the host plant and death [23,26]. *R. solanacearum* is difficult to eradicate because it can survive in soils for many years and uses weeds as a temporary host [27,28]. Breeding for resistant cultivars is the best strategy for the control of *R. solanacearum*. Many studies are attempting to understand the defense mechanisms of plants against *R. solanacearum*. In *Arabidopsis,* resistance is conferred by two resistance genes, the recessive *RRS1-R* (Resistance to *Ralstonia solanacearum* 1) coding for a receptor TIR-NB-LRR protein harboring a C-terminal WRKY DNA binding domain, and RPS4 (Resistance to *Pseudomonas syringae* 4) coding for another R protein [29,30,31,32]. RRS1-R physically associates with RPS4 and together recognize PopP2, a type III effector of *R. solanacearum* [32]. The transfer of the RRS1/RPS4 pair of R genes from *A. thaliana* into tomato was able to confer immunity to *R. solanacearum* [31,33]. The NLR protein Roq1 (Recognition of XopQ1) identified in *Nicotiana benthamiana* recognized the effector proteins XopQ and HopQ1 from *Xanthomonas* and *P. syringae*. Tomatoes expressing Roq1 conferred resistance to *Xanthomonas*, *P. syringae*, and *Ralstonia* [33].

Tomato (*Solanum lycopersicon*) is one of the most important vegetable crops for which annual global production was around 188 million tonnes in 2018 (Global tomato industry report). Bacterial wilt disease may cause up to 90% loss of tomato yield in tropical and subtropical regions of the world [34]. The tomato cultivar Hawaii7996 has been shown to be the most resistant line against various *R. solanacearum* strains [35,36]. However, the defense mechanisms of tomato against *R. solanacearum,* particularly in the early stages of infection, are still unexplained. No R-gene in response to *R. solanacerum* has been identified in tomatoes. Genetic analysis has shown that resistance in tomatoes is polygenic and controlled by several quantitative trait loci (QTLs) including two major loci (Bwr-12, Bwr-6) located on chromosomes 12 and 6 and three minor loci (Bwr-3, Bwr-4, Bwr-8) [37,38,39]. Resistance in tomatoes came from the ability to restrict the spreading of the bacteria from the primary xylem to the meta-xylem and to suppress the numbers of bacteria, which are significantly reduced in the stems of resistant plants [40,41]. Proteomic analyses using classical 2D SDS-page showed no differentially regulated proteins in resistant plants in response to *R. solanacearum* [42]. 

In this work, a comparative phosphoproteomics investigation of resistant and susceptible tomato cultivars after inoculating with *R. solanacearum* was analyzed using LC-MS. A total of 969 phosphoproteins were identified. Specifically, 886 phosphoproteins were detected in Hawaii7996 treated with *R. solanacearum*. Nine proteins were uniquely presented in infected Hawaii7996. Among these, six were characterized. These results provide information on the defense mechanisms in the early events of *R. solanacerarum* infection in tomatoes.

## 2. Results

### 2.1. Bacterial Inoculation and Plant Symptom Development

**Figure 1:**
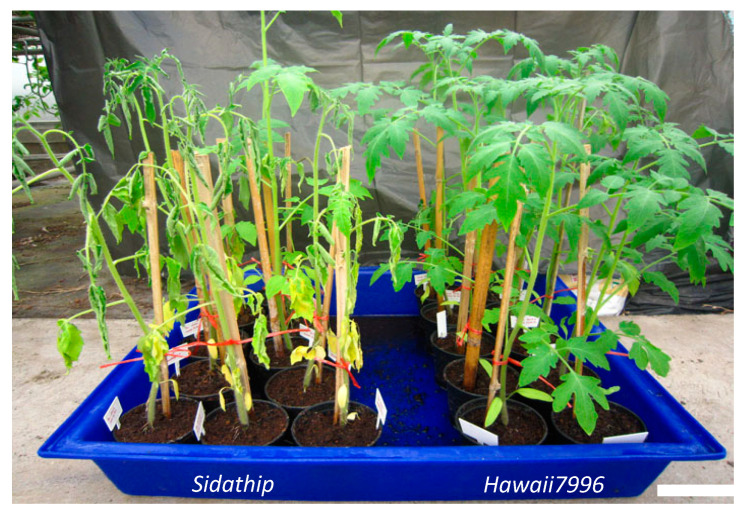
Susceptible tomatoes cv. Sidathip and resistant tomatoes cv. Hawaii7996 inoculated with *R. solanacearum* and symptoms development. Susceptible plants showed wilt symptoms after 7 dpi whereas the resistant Hawaii7996 showed no symptoms. Bar = 10 cm.

Individuals of the resistant tomato cv Hawaii7996 and susceptible tomato cv Sidathip were inoculated with a suspension of *R. solanacearum* (1.0 × 10^7^ colony-forming units [CFU]/mL) by pouring 25 mL of the suspension directly onto cut roots. Ten biological replicates of plants were used for the virulence test. Inoculated tomatoes showed significantly different responses after bacterial inoculation. In susceptible plants, Sidathip started to wilt at 4 dpi (days post-inoculation) and completely wilt after 7 dpi. Hawaii7996 plants infected with *R. solanacearum* did not show any wilt symptoms after 30 dpi (Figure 1).

### 2.2. Analyses of the Stem Phosphoproteome among Resistant and Susceptible Tomatoes

**Figure 2:**
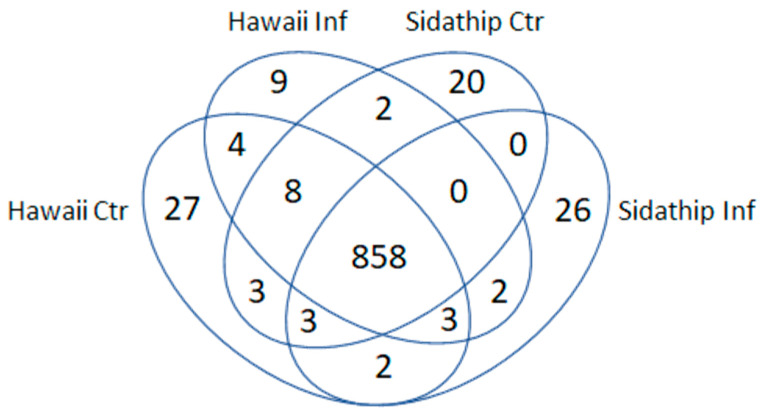
Venn diagram showing overlap pattern of the identified phosphoproteins in the stems of tomato cv. Hawaii7996 inoculated with *R. solanacearum* (Hawaii Inf), tomato cv. Hawaii7996 mock inoculation with sterilized water (Hawaii Ctr), tomato cv. Sidathip inoculated with *R. soalanacearum* (Sidathip Inf), and tomato cv. Sidathip inoculated with sterile water (Sidathip Ctr).

The stems of resistant and susceptible plants were collected at 15 min, 30 min after bacterial inoculation. As a control, plants were cut and inoculated with water. The stems of resistant and susceptible plants by control and treated were collected at 15 min, 30 min from three biological replicates. Total soluble proteins were extracted from collected stems. Then, the phosphoproteins were enriched by the phosphoprotein enrichment kit (Pierce^TM^), tryptic digested, and analyzed by LC-MS. Decyder MS software was used for the quantification of the MS/MS intensity in each sample and peptide sequences were searched against the NCBI protein database using Mascot software identifying 969 phosphoproteins. The overlapping pattern of the phosphoproteins in the four samples was determined by a Venn diagram (Figure 2). From a total of 969 identified phosphoproteins, 858 proteins were present in both cultivars, while 886 and 894 phosphoproteins were detected in resistant Hawai7996 and susceptible Sidathip tomato cultivars treated with *R. solanacearum*, respectively. Further, 910 and 896 phosphoproteins were expressed in Hawai7996 and Sidathip cultivars treated with water, respectively. Nine proteins were particularly common in infected Hawaii7996. Among these, six phosphoproteins were identified: Cinnamyl alcohol dehydrogenase 1 (CAD1), mitogen-activated protein kinase kinase kinase 18 (MAPKKK18), Phospholipase D delta (PLDDELTA), nicotinamide adenine dinucleotide transporter 1 (NDT1), B3 domain-containing transcription factor VRN1 (VRN1), and disease resistance protein RPM1 (RPM1).

### 2.3. Functional Classification of Identified Phosphoproteins

**Figure 3:**
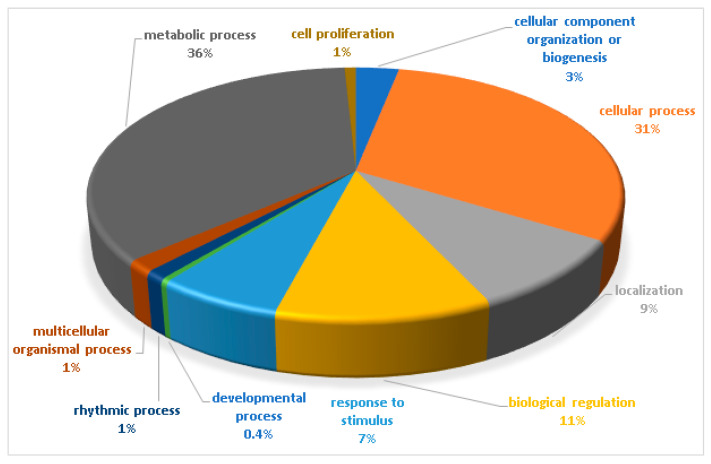
Biological functional classification of the identified phosphoproteins. The percentage of annotated proteins with each indicated gene ontology (GO) term is shown. The biological functions were classified in 10 different groups, mostly functioning in the metabolic process (36%) and the cellular process (31%). The common biological processes included biological regulation (11%), localization (9%), and response to stimulus (7%).

The biological functions of the total identified phosphoproteins were analyzed based on the gene ontology online database Panther16.0 (http://www.pantherdb.org/) (accessed on 12 May 2021). The biological processes are shown in Figure 3. The majority of identified phosphoproteins were involved in the metabolic process (36%), followed by the cellular process (31%). The most common biological processes included biological regulation (11%), localization (9%), and response to stimulus (7%).

### 2.4. Quantitative Real-Time PCR of Phosphoproteins Expressed in Hawaii7996 and Sidathip Treated with R. solanacearum

**Figure 4:**
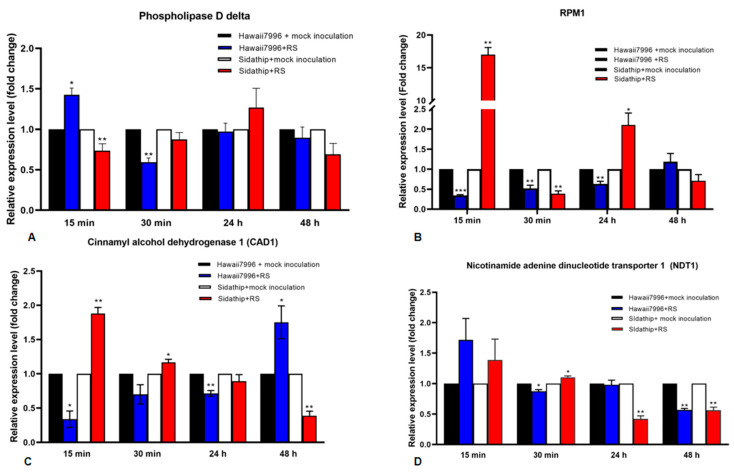
Time course of quantitative Real Time PCR analysis of four genes in susceptible and resistant tomato cultivars, Sidathip and Hawaii7996 inoculated with *Ralstonia solanacearum*. Total RNA was extracted from the stems of resistant tomato cv. Hawaii7996 and susceptible tomato Sidathip at 15 min, 30 min, 24 h, and 48 h after inoculation with *Ralstonia solanacerum* or distilled water for control. The control samples collected from Hawaii7996 and Sidathip treated with water were used for calibration. The level of expression of the genes shows (**A**) phopholipase D delta, (**B**) RPM1, (**C**) cinnamyl alcohol dehydrogenase 1 (CAD1), and (**D**) nicotinamide adenine dinucleotide transporter 1 (NDT1). Error bar represents the standard deviation obtained from three biological replicates. Asterisks indicate the statistical significance level by *p*-value obtained by two tailed student’s *t*-test: *p*-value < 0.05 (*), < 0.01 (**), and < 0.001 (***).

Four out of six genes commonly identified in the resistant cultivar Hawaii7996 inoculated by *R. solanacearum*, which are cinnamyl alcohol dehydrogenase 1 (CAD1), nicotinamide adenine dinucleotide transporter 1 (NDT1), phospholipase D delta (PLDDELTA), and disease resistance protein RPM1 (RPM1), were selected for further function investigation. The expressions of these genes were analyzed by qRT-PCR as shown in Figure 4.

The expression level of Phospholipase D delta (PLDDELTA) was significantly upregulated in Hawaii7996 in the first 15 min, but downregulated after 30 min. In susceptible plants, the PLDdelta gene was downregulated at 15 min. The gene revealed no significant changes in gene expression in Hawaii7996 and Sidathip at 24 h and 48 h (Figure 4A).

The disease resistance gene RPM1 was strongly upregulated in Sidathip, but significantly downregulated at 30 min. RPM1 was downregulated in Hawaii 7996 at 15 min to 24 h (Figure 4B).

The gene encoding CAD1 was significantly upregulated in non-resistant Sidathip after 15 min challenged with *R. solanacearum*, but decreased gradually in the following 30 min to 48 h. In contrast, in the resistant cultivar Hawaii7996, the CAD1 gene was downregulated in 15 min to 24 h, but the mRNA levels steadily increased and were significantly upregulated at 48 h (Figure 4C).

The nicotinamide adenine dinucleotide transporter 1 (NDT1) gene was not statistically significant at the expression level in both Sidathip and Hawaii7996 at 15 min. At 30 min after inoculation, the NDT1 gene was significantly downregulated in Hawaii7996, but upregulated in Sidathip (Figure 4D).

### 2.5. Protein Chemical Interaction Network

**Figure 5:**
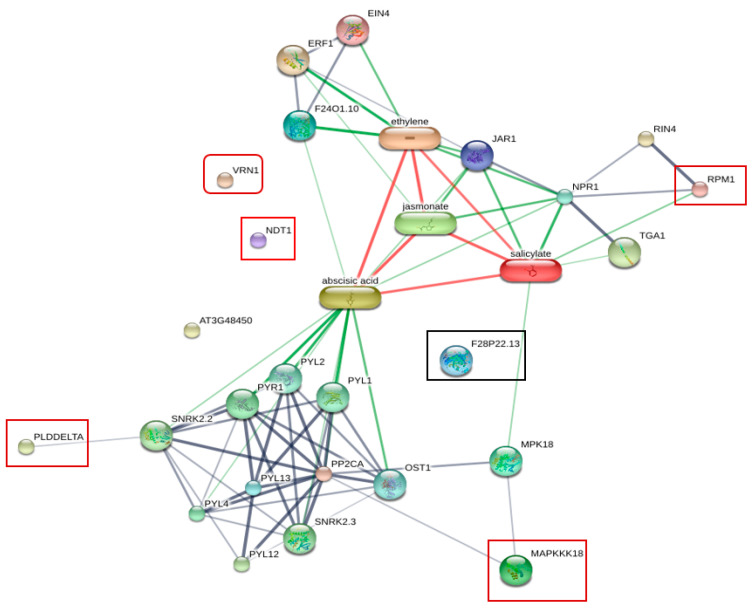
Protein interaction network generated by STITCH 5.0 displaying the association between six phosphoproteins identified from resistant plants inoculated with *R. solanacearum* and plant hormones. RPM1 showed strong association with SA through NPR1 in the protein network. Phospholipase D delta (PLDDELTA) and MAP3K showed associations with abscisic acid. Nicotinamide adenine dinucleotide transporter 1 (NDT1), cinnamyl alcohol dehydrogenase 1 (CAD1 or F28P22.13), and VRN1 showed no network interaction.

To obtain information about the protein–protein interaction network or protein chemical interaction between six identified phosphoproteins, including CAD1, VRN1, NDT1, PLDdelta, MAPKKK (MAP3K), RPM1, and plant hormones, we used the online database of STITCH 5.0 for analysis (Figure 5). The association between some of these proteins and plant hormones including abscisic acid (ABA), salicylic acid (SA), jasmonate acid (JA), and ethylene (ET) was observed. SA is the main plant defense hormone involved in the regulation of plant immunes PTI and ETI [43]. SA-mediated resistance is effective against biotrophic pathogens, but ineffective against necrotrophic pathogens [44], whereas ET/JA-mediated resistance is effective against necrotrophic pathogens. However, hormonal crosstalk between the SA pathway and the ET/JA Pathway has been observed [45]. RPM1 showed a strong association with SA through NPR1, a regulator of systemic acquired resistance (SAR), in the protein network. MAP3K (MAPKKK18) was linked to SA through MPK18. The activation of MAPK induced signaling cascades [46]. In Arabidopsis, MPK18 is a regulator of the functions of microtubules.

Abscisic acid (ABA) is involved in the responses against abiotic stresses and biotic stress. In *Arabidopsis*, ABA induces resistance against *Pythium irregulare* [47]. The functions of ABA in plant defense are varied, including closing stomata and the accumulation of callose at the cell wall. However, many reports show that ABA plays antagonistic roles to SA and ET [48,49]. Phospholipase D delta (PLDDELTA) showed associations with ABA. PLD delta is involved in ROS production and ABA signaling of guard cells [50]. Nicotinamide adenine dinucleotide transporter 1 (NDT1), cinnamyl alcohol dehydrogenase 1 (CAD1 or F28P22.13), and VRN1showed no interaction in this network.

## 3. Discussion

Bacterial wilt is one of the most devastating plant diseases, causing severe loss of crop yield up to 90% [51]. It has a wide host range across over 450 species including those of economic importance such as tomato, potato, tobacco, and banana. The wilt symptom develops by the invasion of the bacteria into the xylem vessel by breaking the vessel walls via cellulolytic enzymes. Within the xylem, the bacteria can spread throughout the plant, and approach high densities leading to the accumulation of exopolysaccharides, which clog vascular vessels [23]. In tomato, Hawaii7996 is the most effective cultivar showing strong resistance against *R. solanacearum* and is used as rootstocks for susceptible commercial lines [52]. By QTL mapping, resistance comes from the two major quantitative trait loci (QTLs) located in chromosomes 12 and 6 (Bwr-12 and Bwr-6) and the three minor loci (Bwr-3, Bwr-4, and Bwr-8) [39]. The model of resistance mechanisms points to the abilities of plants to inhibit the invasion of bacteria through four mechanisms including limiting bacterial entry to the root, vertical movement to the stem, dispersal from vessel to vessel, and spreading from the xylem into the pith or cortex tissue [52]. The number of bacteria has been shown to be suppressed in the stems below the first leaf of the resistant tomato cv. LS-89 [53], indicating the stems have unknown mechanisms that can limit bacterial expansion. Electron microscopy of the LS-89 stems revealed the accumulation of electron-dense materials around the pits and parenchyma cells adjacent to the vessels [40,41]. Moreover, by light microscopy, the bacteria are presented in the primary xylem of resistant plants, whereas in non-resistant plants, they are found in xylem and pit tissue [54]. Therefore, we used the stems for the phosphoproteomics analysis to reveal the early response mechanisms. Protein phosphorylation is a post-translation modification process, which plays an important role in the regulation of signaling cascades in many cell processes including plant defense responses against biotic and abiotic stress [3]. Plant defense mechanisms against pathogens include the production of antimicrobial compounds, lignification of cell walls, the hormone signaling network, the expression of PR (pathogenesis-related) gene, and the hypersensitive response (HR). In this study, we performed a comparative phosphoproteomics analysis of the stems of resistant Hawaii7996 and susceptible Sidathip in response to *R. solanacearum* using LC-MS. During virulence testing, the non-resistant Sidathip completely wilted at 7–8 dpi, but the resistant Hawaii7996 did not wilt by 30 dpi and was able to bear fruits. Phosphoproteomic analysis showed the identification of 969 regulated phosphoproteins belonging to 10 functional categories, providing some information about signal transduction during the initial stages of *R. solanacearum* interaction. Most of the identified phosphoproteins were shared by both cultivars; however, we identified six unique phosphoproteins in the resistant cultivar Hawaii7996: Cinnamyl alcohol dehydrogenase 1, Mitogen-activated protein kinase kinase kinase 18, Phospholipase D delta, Nicotinamide adenine dinucleotide transporter 1, B3 domain-containing transcription factor VRN1, and Disease resistance protein RPM1. Among these, we selected four genes for gene expression analysis by qRT-PCR.

Cinnamyl alcohol dehydrogenase (CAD) is an essential enzyme in the biosynthesis of monolignol, the main component of lignin [55]. Lignin is the major component of the secondary cell wall and functions as a physical barrier to pathogen invasion. Lignin has been demonstrated to be deposited at the sites of the HR region during pathogen infection [56]. In this work, phosphorylated CAD1 was detected in the resistant tomato Hawaii7996 inoculated with *R. solanacearum*. Surprisingly, by gene expression analysis through qRT-PCR, the mRNA level of the CAD1 gene was highly upregulated in Sidathip, whereas this was downregulated in Hawaii7996 at 15 min to 30 min after inoculation. After 48 h, the level of mRNA in Sidathip was significantly lower than that in Hawaii7996. In the early minutes of inoculation, the phosphorylated CAD1 proteins in resistant Hawaii7996 possibly originate from pre-existing proteins and not from freshly synthesized proteins. In the non-infected condition, the stem of Hawaii7996 was physically stronger and more upright than susceptible plants. The CAD1 was probably presented constitutively in minimal quantities in Hawaii7996. In wheat, *Triticum monococcum*, based on microarray-based comparative transcriptomics, showed a differential expression pattern of a wheat CAD gene, TaCAD12, and was more highly expressed in resistant wheat lines than in susceptible plants after inoculation with the necrotrophic fungus *Rhizoctonia cerealis* that causes sharp eyespot disease at 4 days after inoculation. The knockdown mutant of the *TaCAD12* gene resulted in susceptibility to *R. cerealis*, whereas overexpression of this gene enhanced resistance to this fungus. Moreover, silencing of the CAD gene enhances the susceptibility of leaf tissues to the fungal pathogen *Blumeria graminis* f. sp. *Tritici,* causing the powdery mildew disease [57].

Nicotinamide adenine dinucleotide transporter 1 (NDT1) is a nicotinamide adenine dinucleotide (NAD) carrier, which belongs to the mitochondrial carrier family (MC). We found phosphorylated NDT1 uniquely in resistant plants of Hawaii7996. In *Arabidopsis*, there are 58 membrane proteins in the MC family. Among these, three proteins were characterized: AtNDT1, ATNDT2, and AtPXN. AtNDT1 and AtNDT2 were located in the mitochondrial membrane and function as carriers that uptake NAD+ into mitochondria [58]. AtPXN was located on the peroxisomal membrane [59,60]. Recently, the function of NDT as being directly associated with defense mechanisms has been rarely discussed. However, these proteins import NAD, a pyridine nucleotide, which functions as an electron acceptor in oxidation-reduction reactions and coenzyme for many metabolic processes [61,62]. NAD plays a role in cell signaling in plants, animals, and fungi [63]. In *Arabidopsis*, the knockdown mutant of the aspartate oxidase (AO), the first enzyme of NAD synthesis in the chloroplast, caused the loss of stomata immunity against the hemibiotrophic pathogen *Pseudomonas syringae* pv tomato. Exogenous application of NAD induced resistance in many plants. In *Arabidopsis*, extracellular application of NAD induced *PR* gene expression, increasing resistance against *Pseudomonas syringae* [64]. Moreover, in citrus plants, exogenous NAD induced disease resistance to citrus canker, *Xanthomonas citri* susp. *Citri* [65].

Phospholipase D (PLD) is a family of phospholipase enzymes, which hydrolyzes membrane lipids into phosphatidic acid (PA) and a head group. PA is a secondary messenger, which is involved in the regulation of many cellular processes including senescence, seed germination, and abiotic and biotic stress [66,67]. The complexity of plant PLD functions reflects the number of PLD members in different organisms. The *Arabidopsis* genome contains 12 PLD isoforms subdivided into three α-, two β-, three γ-, one δ-, one ε-, and two ς-class isoforms [67], whereas two PLD genes are found in mammals, and one PLD in yeast [66]. In this work, we found the phosphorylated PLDdelta protein in resistant tomatoes Hawaii7996 at 15 min after inoculation. qRT-PCR expression analysis showed that PLDdelta was upregulated in resistant Hawaii7996 after inoculation, but the level of expression decreased after 30 min. Recent reports show that PLDdelta is involved in the defense response against phytopathogens. PLDdelta is activated by oleic acid and is associated with microtubules [67,68]. Activated PLDdelta decreased H_2_O_2_ leading to an anti-hypersensitive response (anti-HR) effect [69]. HR is an important plant defense mechanism, which confines pathogens to the initial invasion area and not to spread to other tissues. However, HR is effective against biotrophic pathogens, whereas it is non-effective against necrotrophic pathogens. The necrotrophic pathogens induce HR to spread into the dead tissues [70]. PLDdelta possibly reduced the HR level in the resistant Hawaii7996 to prevent the distribution of *R. solanacearum*. In *Arabidopsis*, the PLDdelta-GFP fusion protein localized to the plasma membrane at the attack site of *Blumeria graminis* f. sp. *hordei* (Bgh) and the plants with the knockout mutant of *PLDdelta* increased susceptibility to Bgh, indicating PLDdelta was involved in resistance against barley powdery mildew fungi into epidermal cells [71]. In rice, PLDdelta was observed at the plasma membrane, closed to the attack site of *Xanthomonas oryzae* pv *oryzae* [72].

*RPM1* is a NOD-like receptor (NLR), an R gene product identified in *A. thaliana*, which confers resistance to *P. syringae* expressing either of two effector proteins, *avrRpm1* or *avrB*. The recognition of the effectors activates RPM1 resulting in downstream signal transduction including the influx of extracellular Ca^2+^, ROS production, the accumulation of phosphatidic acid (PA), and HR response at the infection site [73,74]. Using LC-MS analysis, we found phosphorylated protein RPM1 in resistant tomatoes cv. Hawaii7996, but no phosphorylated RPM1 was detected in susceptible tomatoes cv. Sidathip. By qRT-PCR analysis, the level of RPM1 expression in Sidathip was highly upregulated at 15 min after inoculation. However, the expression was significantly downregulated after 30 min. By contrast, the level of RPM1 in Hawaii7996 was downregulated after 15 min inoculation. The results implicated the high level of inactive RPM1 in Sidathip. The mechanism involved in the induction of RPM1 gene expression in Sidathip is unknown. The plant defense hormone SA, which is involved in the response against biotrophic pathogens, has been shown to induce the expression of different R-genes. SA treatment induced TaRPM1 expression in wheat [75]. Exogenous application of SA induced wheat R gene TaRGA, conferring the resistance against wheat powdery mildew *Blumeria graminis* f. sp. Tritici [76]. Phosphorylated RPM1 in Hawaii7996 possibly mediated ROS production to strengthen the cell wall via the cross-linking of glycoproteins. An accumulation of ROS leads to the induction of HR. The necrotrophic *R. solanacearum* possibly induced the expression of RPM1 in the susceptible tomatoes to trigger the downstream HR to promote the infection.

## 4. Conclusions

In this study, we performed Phosphoproteomics analyses to obtain information on the activated proteins in tomatoes against *R. solanacearum*, thereby a total of 969 phosphoproteins was identified. Nine phosphoproteins were uniquely detected in resistant Hawaii7996, and among these, four phosphoproteins were analyzed at the transcriptional level using qRT-PCR. PLDdelta, NDT1, CAD1, and RPM1 have been reported to be involved in the defense response against various phytopathogens. However, the expression patterns of these genes are different. PLD delta involved in the defense signaling was significantly upregulated in resistant plants at 15 min after inoculation. Surprisingly, the expression of receptor protein RPM1 is strongly upregulated in susceptible plants at 15 min after inoculation indicating that susceptible plants mediated different defense hormone pathways than resistant plants. In the future, the association between plant hormones and these proteins could be investigated. The information obtained in this work provides clues to understand the early resistant mechanisms of tomatoes against *R. solanacearum*.

## 5. Materials and Methods

### 5.1. Plant Materials and Growth Conditions

Seeds of the resistant Hawaii7996 (*Solanum lycopersicum*) and susceptible Sidathip (*Solanum lycopersicum*) tomato lines were obtained from Dr. Chalida Leksombun, Kasetsart University, Thailand. Seeds were surface sterilized with 30% bleach for 5 min and washed with sterile distilled water. The seedlings were germinated in sterile wet tissue closed in a plastic bag for 5 days. Each seedling was then transferred to an individual pot with approximately 250 g of autoclaved soil and grown in a climate chamber (30/28 °C day/night temperature, 12 h photoperiod, sunlight).

### 5.2. Ralstonia Solanacearum Strains and Inoculum Preparation

*R. solanacearum* Ka21 (race 1, biovar 3) inoculates were obtained from Dr. Chalida Leksombun, Kasetsart University, Thailand. The bacteria were grown in a tetrazolium chloride medium (TTC; 0.01% casaminoacids, 1% peptone, 1% glucose, and 0.005% tetrazolium chloride) at 28 °C for 2 days to identify virulent colonies. A single virulent colony was then grown on CPG medium (0.01% casaminoacids, 1% peptone, 1% glucose, and 1.5% Agar) for 48 h at 30 °C. The bacterial inoculum was prepared by scraping the cultured bacteria into 20 mL sterile water and adjusting the concentration to an OD_650_ of 0.1 (2 × 10^7^ CFU/mL) in 1 L sterile water. The control inoculum contained sterile water with no addition of bacteria. Four-week-old plants were inoculated by pouring the above inoculum to reach a final concentration of approximately 10^7^ CFU/g of soil. Three replicates were performed for both *R. solanacearum* and mock inoculation.

### 5.3. Total Protein Extraction

Three biological replicates of control and *Ralstonia solanacearum* inoculated stems were collected at 15 min and 30 min after inoculation. Total proteins were extracted and quantified according to the method of Lowry (1951) using BSA as the protein standard. Approximately 0.5 g of fresh stems from each biological replicate was ground into a fine powder in liquid nitrogen in a mortar and solubilized with 0.5% SDS solution. The supernatant was transferred to a new tube, mixed well with 2 volumes of cold acetone, and incubated overnight at −20 °C. After centrifugation at 10,000× *g* for 15 min, the pellet was dried and stored at −80 °C before use.

### 5.4. Phosphoprotein Enrichment

Phosphoproteins were enriched following the protocol provided in the phosphoprotein enrichment kit user manual (Pierce, Thermo Scientific, Waltham, MA, USA). Briefly, the protein pellet was dissolved in Lysis-Binding-Wash Buffer containing CHAPS, protease inhibitor, and a phosphatase inhibitor, and then mixed vigorously for five minutes before centrifugation at 10,000× *g* for 20 min at 4 °C. The supernatant was subjected to determine the protein concentration using the Lowry protein assay. The protein mixtures were diluted with Lysis-Binding-Wash Buffer to achieve a final volume of 0.5 mg/mL. The diluted protein mixtures were mixed with a proprietary enrichment gel and then incubated in the column for 30 min at room temperature. The resin was washed 3 times with Lysis-Binding-Wash Buffer containing CHAPS provided in the kit to remove unbound proteins. Finally, the phosphoproteins were eluted from the resin by elution buffer, and the process was repeated four more times. The collected phosphoprotein solutions were desalted, and the resulting solutions were further subjected to concentrate by Speed Vac centrifugation.

### 5.5. Phosphoprotein Digestion

Five micrograms of bacterial phosphoprotein samples were subjected to in-solution digestion. Samples were completely dissolved in 10 mm ammonium bicarbonate (AMBIC), disulfide bonds were reduced using 5 mm dithiothreitol (DTT) in 10 mm AMBIC at 60 °C for 1 h, and sulfhydryl groups were alkylated using 15 mm Iodoacetamide (IAA) in 10 mM AMBIC at room temperature for 45 min in the dark. Then, samples were mixed with 50 ng/µL of sequencing-grade trypsin (Promega, Walldorf, Germany) and incubated at 37 °C overnight. Prior to LC-MS/MS analysis, the digested samples were dried and redissolved with 0.1% formic acid.

### 5.6. LC-MS Analysis

LC-MS/MS analysis of digested peptide mixtures was performed using a Waters SYNAPT™ HDMS™ system. The 1D-nanoLC was carried out with a Waters nano ACQUITY UPLC system. Four microliters of digests was injected onto the RP analytical column (20 cm × 75 μm) packed with a 1.7 μm Bridged Ethyl Hybrid (BEH) C18 material (Waters). Tryptic peptides were eluted with a linear gradient from 2% to 40% acetonitrile developed over 60 min at a flow rate of 350 mL/min. This was followed by a 15 min period of 80% acetonitrile to clean the column before returning to 2% acetonitrile for the next sample. The effluent samples were electrosprayed into a mass spectrometer (Synapt HDMS) for MS/MS analysis of peptides. Argon gas was used in the collision cell to obtain MS/MS data. MS/MS spectra thus obtained were processed using Max Ent 3, deconvolution software for peptides (Ensemble 1, Iterations 50, auto peak width determination) within Mass Lynx 4.0. The protein spectral data used in this study have been deposited at ProteomeXchange: PXD030242 and JPST001417.

### 5.7. Proteins Quantitation and Identification

For protein quantitation, DeCyder MS Differential analysis software (DeCyderMS 2.0, GE Healthcare) [77,78] was used. The analyzed MS/MS data from DeCyderMS were submitted to a database search using Mascot software (Matrix Science, London, UK, [79]). The data were searched against the NCBI database for protein identification. Database interrogation was taxonomy (*Solanum lycopersicon*); enzyme (trypsin); variable modifications (carbamidomethyl, oxidation of methionine residues); mass values (monoisotopic); protein mass (unrestricted); peptide mass tolerance (1.2 Da); fragment mass tolerance (±0.6 Da), peptide charge state (1+, 2+ and 3+), max missed cleavages (1), and instrument = ESI-Q-TOF. The relative quantitation ratios were displayed as log2 and processed with median normalization for each sample. Proteins considered as identified proteins had at least one peptide with an individual mascot score corresponding to *p* < 0.05.

### 5.8. Bioinformatics Analysis

Data normalization and quantification of the changes in protein abundance between the control and treated samples were performed and visualized using *MultiExperiment Viewer* (Mev) software version 4.6.1 [80]. Briefly, peptide intensities from the LC-MS analyses were transformed and normalized using a mean central tendency procedure. Green, black, and red colors represent proteins with low, average, and high levels of expression, respectively. Gene ontology annotation including the biological process, cellular component, and molecular function was performed by using Panther [81]. Jvenn, a plug-in for the jQuery Javascript library, was used to visualize the number of all differentially abundant proteins present in each group and their intersections among the different sample groups [82]. The identified proteins were simultaneously submitted to the Search Tool for Interacting Chemicals (STITCH) (http://stitch.embl.de) (accessed on 12 May 2021) to search for an understanding of cellular functions and the potential interaction between protein–protein and protein–chemical interactions as shown in Figure 5 [83].

### 5.9. Real-Time Quantitative PCR Expression Analysis

The stems of resistant and susceptible tomato cultivars inoculated with *R. solanacearum* or distilled water were sampled by dissecting the stems at 15 min, 30 min, 24 h and 48 h after inoculation. Each sample consisted of the pooled stems derived from three plants. Total RNA from the stems was isolated using the TRIzol reagent (Invitrogen, Carlsbad, CA, USA) according to the manufacturer’s protocol. The quantity and quality of total RNA were examined on 1% agarose-formaldehyde RNA gel by electrophoresis. The total RNA was treated with DNase I (Thermo Fischer Scientific, USA) to remove the DNA. The RNA from each sample was adjusted to have an equivalent concentration using Nanodrop and then reverse-transcribed to cDNA using Maxima reverse transcriptase (Thermo Fischer Scientific, USA) and the Oligo dT Primer in accordance with the manufacturer’s instructions. After the reaction, the samples were 10-fold diluted and used as template DNA for real-time quantitative PCR. Real-time PCR was performed using Bioneer (EXicycle Tm 96) and 5x Hot Firepol EvaGreen qPCR Mix Plus (Solis BioDyne). Gene-specific primers were designed using OligoAnalyzer 3.1 (Integrated DNA Technologies, Singapore) and Primer3Plus. The primers of genes of interest were the following: (CAD1, protein accession nr. 460369778) CAD1-F: 5′-CCAACCCAAGGTGGTTTTGC-3′, CAD1-R: 5′-CCCATGTGTCCAACTCCTCC-3′; (PLDdelta, accession nr. 460373442), PLD-F: 5′-CACGACGAGGAAACCAGGAA-3′, PLD-R: 5′-TGCCTGCGTATCAACCAGAA-3′; (NDT1 accession nr. 460386444), NDT1-F: 5′-AAGTTGGTGCTGGTGCTTCT-3′, NDT1-R: 5′-GGCCTCGATAAAGTCCCCTG-3′; (RPM1 accession nr. 460397731), RPM1-F: 5′-ATGGAATCACCGAGCTGCAT-3′, RPM1-R: 5′-TGCTATATCCGCGTTTGCCT-3′. Expression values were normalized using *ACT2* (Actin2: SGN-U580609, [84]). ACT2-F: 5′-AGATGGGGCTATGAAAGAAGG-3′; ACT2-R: 5′-AACAACACAATCACTCTCCG-3′). These genes were amplified in three replicates under the following conditions: 95 °C for 3 min, initial denaturation; 95 °C for 30 s, 60 °C for 30 s, 34 cycles. The relative quantification of specific mRNA levels was performed using the delta Ct method (2^(−ΔCt)^ method) [85]. Data were analyzed with Student’s *t*-test in Graphpad prism 9.0.

## Data Availability

The data is contained within this article.
